# Off-Flavor Compounds in Fermented Cocoa: Impact of Overfermentation

**DOI:** 10.1021/acs.jafc.6c05685

**Published:** 2026-07-14

**Authors:** Franziska Krause, Martin Steinhaus

**Affiliations:** † Technical University of Munich, TUM School of Natural Sciences, Department of Chemistry, Lichtenbergstraße 4, 85748 Garching, Germany; ‡ Leibniz Institute for Food Systems Biology at the Technical University of Munich (Leibniz-LSB@TUM), Lise-Meitner-Straße 34, 85354 Freising, Germany

**Keywords:** Theobroma cacao L., fermentation time, butanoic acid, 2-methylpropanoic acid, 2-methylbutanoic acid, 3-methylbutanoic acid, 2-methoxyphenol, 4-ethylphenol, odor activity value (OAV)

## Abstract

The compounds responsible for a variety of off-flavors that occasionally occur in fermented cocoa have recently been identified, but their origin is not yet fully understood. However, overfermentation has been suggested as a possible source. Quantitation of potential off-flavor compounds in well fermented and overfermented samples resulting from fermentation trials in Nicaragua and Costa Rica showed that overfermentation can lead to smoky, leathery, and horse stable-like off-flavors caused by 2-methoxyphenol and 4-ethylphenol, and to cheesy, vomit-like off-flavors caused by short-chain carboxylic acids such as 2-methylpropanoic acid, butanoic acid, 2-methylbutanoic acid, and 3-methylbutanoic acid. The concentrations of 2-methoxyphenol and 4-ethylphenol in the overfermented cocoa samples were in a similar range as previously reported in cocoa samples with wood smoke contamination during artificial drying. To avoid overfermentation, measuring the pH of the fermentation mass emerged as a potential alternative to the daily evaluation of a representative number of cocoa bean cross sections.

## Graphical abstract

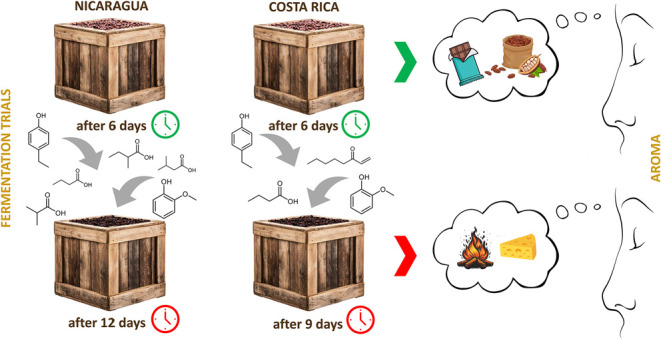

## 1 Introduction

Cocoa processing is traditionally divided into two steps: In tropical regions, cocoa producers grow cocoa trees (*Theobroma cacao* L.), harvest the fruits, and extract, ferment, and dry the seeds commonly known as cocoa beans. Subsequently, and predominantly in temperate regions, the fermented cocoa beans are processed into chocolate and other cocoa products. The major cocoa cultivation region today is West Africa, followed by South America and Southeast Asia. Cocoa trees reach heights of 5–15 m and bear fruit on the trunk and large branches. After harvest, the fruits, commonly called pods, are opened to obtain the cocoa beans with the adherent sugary pulp.^1–3^ This material is commonly placed in boxes or arranged in heaps to initiate fermentation. Spontaneous fermentation by microorganisms from the local environment is the norm, whereas the application of defined starter cultures is still rare. The fermentation time is highly variable and depends on factors such as the weather, the cocoa variety, the size of the fermentation boxes, and the individual fermentation protocol, but it is usually in the range of 3 to 8 days. During the first 24 h, yeasts convert sugar into ethanol. In this phase, the pulp fills the cavities between the beans and ensures anaerobic conditions. Subsequently, decomposition of the pulp in combination with mixing of the beans initiates the second, aerobic phase, in which predominantly acetic acid bacteria and lactic acid bacteria convert ethanol into organic acids. The exothermic formation of acetic acid dominates and leads to an increase in the temperature of the fermentation mass. Temperatures above ∼45 °C in combination with acetic acid penetrating the testa kill the embryo and thus prevent germination.^4,5^ Intracellular membranes are degraded, which initiates a multitude of biochemical reactions. Polyphenol oxidases degrade polyphenols, which changes the color of the beans from violet to brown and substantially reduces bitterness and astringency.^2,4–7^ The sharp drop in pH promotes protein degradation by proteases, resulting in the formation of amino acids and peptides as important flavor precursors.^6–11^

The fermentation process is highly variable. Therefore, reliable decision criteria are required to determine when to switch from fermentation to drying. This point in time is often solely determined by the color, temperature, and smell of the fermentation mass. Cut tests, in which the degree of fermentation is assessed based on cross-sectional cuts of the beans, allow better control of the process.^12^ After fermentation, the cocoa beans are dried to <8% moisture to inhibit further microbial activity. Sun drying is common; however, artificial drying by hot air is also frequently applied.

Fermented cocoa is occasionally tainted with off-flavors that can persist during further processing and then substantially reduce the quality of chocolate and other cocoa products. Recent studies have provided a comprehensive overview of the compounds responsible for typical cocoa off-flavors. 2-Methoxyphenol, 3- and 4-methylphenol, 3- and 4-ethylphenol, and 2,6-dimethoxyphenol can cause smoky off-flavors.^13,14^ Geosmin often accounts for moldy and earthy off-flavors.^15^ Oct-1-en-3-one may evoke mushroom-like off-flavors.^13,16,17^ The carboxylic acids acetic acid, 2-methylpropanoic acid, butanoic acid, 2-methylbutanoic acid, and 3-methylbutanoic acid were associated with putrid, cheesy, rancid, fecal, pungent, and sweaty off-flavors in cocoa.^9,18,19^ However, little is known about the process conditions that lead to the formation of these off-flavor compounds and must therefore be avoided in the production of high-quality cocoa. As the only exception, a recent study revealed wood smoke contact during drying of cocoa as a source of the above-mentioned, odor-active phenols.^14^ Nevertheless, overfermentation is discussed as an alternative source of these smoky compounds, and overfermentation has also been associated with the formation of cheesy-smelling carboxylic acids.^20^

Overfermentation can occur if the optimal fermentation time is exceeded. Characteristic signs of overfermentation are an increase in pH, a decrease in temperature, blackening of the beans, and the development of off-flavors. These changes are associated with changes in the microbial flora.^9,20,21^ Only three previous studies have addressed the overfermentation of cocoa beans. Based on the results of sensory tests, Biehl et al. suggested that desirable odor-active compounds were degraded during overfermentation while undesirable odorants were formed.^21^ Oberparleiter and Ziegleder reported elevated concentrations of banana-like smelling 3-methylbutyl acetate in an overfermented cocoa.^22^ In an elaborate study, Lopez and Quesnel showed that particularly an elongated aerobic phase led to off-flavors caused by high levels of propanoic acid, 2-methylpropanoic acid, butanoic acid, and 3-methylbutanoic acid.^20^

In summary, the role of overfermentation for the formation of odorants causing off-flavors in cocoa is poorly understood. To fill this gap, we conducted two fermentation trials on-site, one in Nicaragua and one in Costa Rica. The previously identified cocoa off-flavor compounds were quantitated in the dried beans after the optimal fermentation time as a reference and after overfermentation. Their aroma impact was assessed based on odor activity values (OAVs) obtained by dividing the concentrations in the cocoa samples by the odor threshold concentrations (OTCs) in deodorized cocoa butter.

## 2 Materials and Methods

### 2.1 Chemicals

The following reference odorants were purchased from commercial sources: acetic acid, 4-ethylphenol, geosmin, 3-methylphenol, 2-methylpropanoic acid (Merck; Darmstadt, Germany), 2,6-dimethoxyphenol, 3-ethylphenol, 2-methylbutanoic acid, oct-1-en-3-one (Thermo Fisher Scientific; Dreieich, Germany), butanoic acid, 2-methoxyphenol, 3-methylbutanoic acid (TCI; Tokyo, Japan), and 4-methylphenol (ABCR; Karlsruhe, Germany). The following isotopically substituted odorants were synthesized according to procedures from the literature: (^2^H_2_)­butanoic acid,^23^ (^2^H_5–8_)-2,6-dimethoxyphenol,^24^ (^2^H_2_)-4-ethylphenol,^13^ (^2^H_3_)-(−)-geosmin,^25^ (^2^H_3_)-2-methoxyphenol,^26^ (^2^H_2_)-3-methylbutanoic acid,^27^ and (^2^H_2–4_)­oct-1-en-3-one.^28^ (^2^H_3_)­acetic acid and (^2^H_7_)-4-methylphenol were purchased from Merck. (^2^H_9_)-2-Methylbutanoic acid and (^2^H_7_)-2-methylpropanoic acid were purchased from CDN Isotopes (Quebec, Canada). Dichloromethane from CLN (Langenbach, Germany) was freshly distilled through a column (120 cm × 5 cm) packed with Raschig rings. Deodorized cocoa butter was from Cargill (Berlin, Germany).

### 2.2 Cocoa Fermentation in Nicaragua

Ripe and healthy cocoa pods of the local clone mix were harvested from the Ritter Sport cocoa plantation El Cacao in Nicaragua. After a maximum pod storage time of 24 h, beans with adhering pulp were mechanically removed from the pods, and transferred to a wooden fermentation box. The fermentation mass (500 kg) was covered with jute bags. Mixing was performed every 24 h, early in the morning, by manually transferring the fermentation mass to another fermentation box using a shovel. Before mixing, the temperature in the center of the fermentation mass was recorded. After mixing, a representative sample of 60 to 70 beans and a sample of the pulp were taken for daily on-site analyses. The pulp samples collected immediately after the start of fermentation and after 1 day were cut from the collected beans. Subsequent samples were obtained by immersing hands into the fermentation mass and wiping off the adhering pulp. Details on monitoring the fermentation process are available in the Supporting Information file.

### 2.3 Cocoa Fermentation in Costa Rica

Ripe and healthy cocoa pods of the local clone mix were harvested from the cocoa plantation Tres Equis–Finca de Cacao of Rausch PCE, Turrialba. After a maximum pod storage time of 24 h, beans with adhering pulp were manually removed from the pods, and transferred to a wooden fermentation box. The fermentation mass (50 kg) was first covered with a layer of banana leaves and then with a plastic tarpaulin. Every 24 h, early in the morning, the fermentation mass was stirred inside the box using a wooden shovel. Sampling and on-site analysis were carried out in essentially the same manner as in Nicaragua. Details on monitoring the fermentation process are available in the Supporting Information file.

### 2.4 Quantitation Assays

Quantitation of the off-flavor compounds was accomplished by GC–MS in combination with stable isotopically substituted internal standards.^14^ Sample preparation is detailed in the Supporting Information file. All quantitations were carried out in triplicate using three independent sample workups. The GC–MS analyses were performed using a simple GC–MS system with an ion trap mass spectrometer or a two-dimensional heart-cut GC–GC–MS system with a high-resolution Q Exactive GC Orbitrap mass spectrometer. Internal standards, quantifier ions, calibration lines, and individual concentration data used for mean calculations are available in the Supporting Information file, Tables S1, S3, S4, S7, S8, S11, S12, S14, and S15.

## 3 Results and Discussion

### 3.1 Cocoa Samples from Nicaragua: Fermentation Data

Fermentation parameters were adapted to the method commonly practiced on the cocoa farm. This included particularly box type, batch size, and mixing frequency. These parameters would typically result in an optimum fermentation time of 5–6 days. The actual fermentation progress, however, was monitored by evaluation of cross sections of the cocoa beans and by collecting physical and chemical key data.

Figure 1 shows the pods before opening, as well as the fermentation mass and the corresponding cross-sectional images of the beans at fermentation onset, after 6 days, and after 12 days of fermentation. The pods were ripe and showed no signs of disease. The beans, freshly removed from the pods and placed in the fermentation box, showed a whitish pulp firmly sticking to the beans. Clear liquid accumulated and slowly drained through holes in the bottom of the fermentation box. The cross-sectional image showed purple-colored beans of varying shades. The beans had a firm texture.

**Figure 1:**
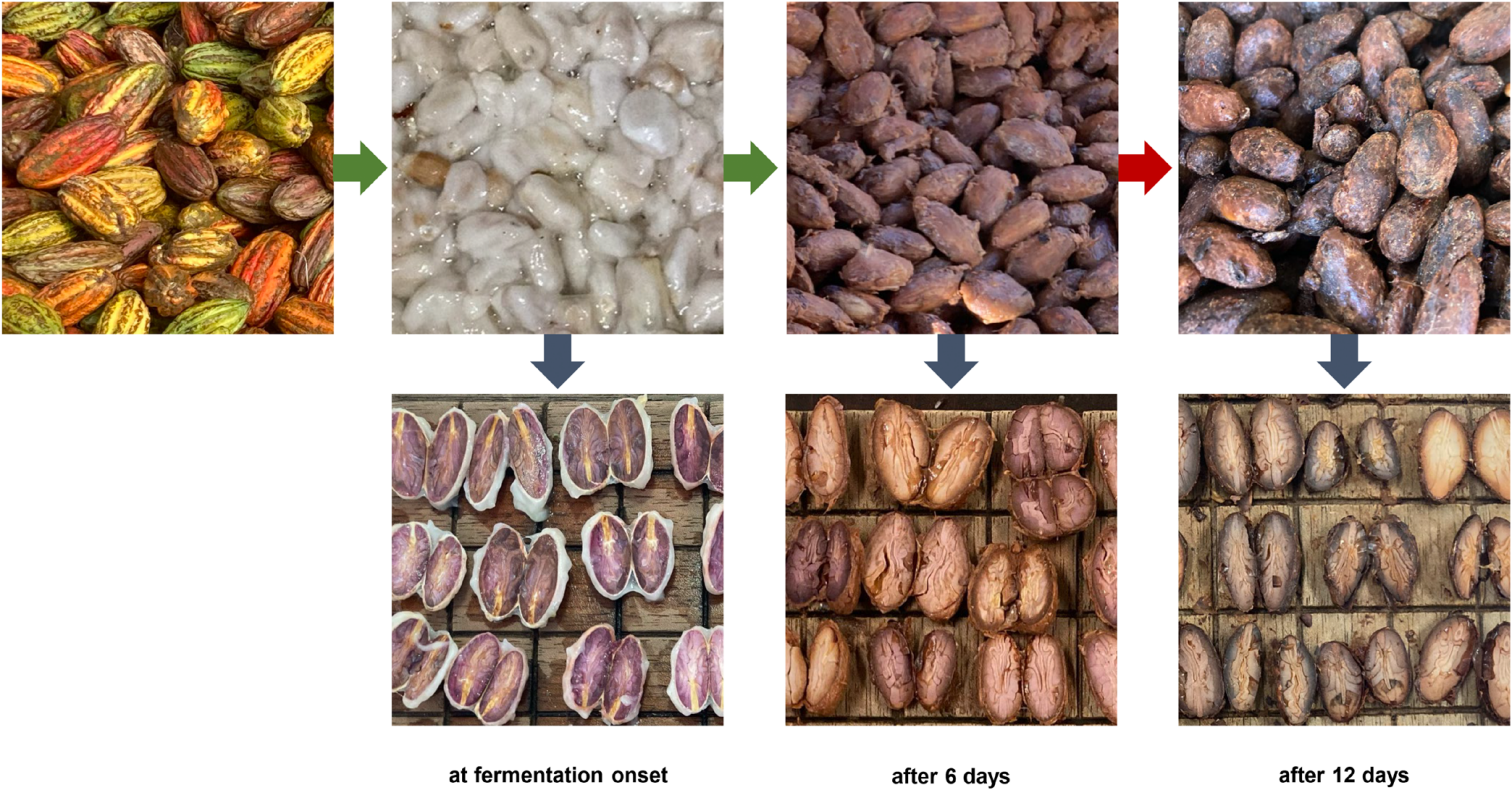
Cocoa fermentation in Nicaragua: cocoa pods before opening, fermentation mass and cross-sectional images on day 0, day 6 (reference sample), and day 12 (overfermented sample).

Figure 2 depicts the changes of physical and chemical key data during fermentation, including temperature and pH as well as sugar, ethanol, and acetic acid concentrations. In addition, the percentage of well-fermented beans was determined by cross-sectional analysis. Detailed data is available in the Supporting Information file, Table S2. In the first phase of the fermentation, the sugar content in the pulp declined sharply, dropping from 49 g/100 g dry matter (DM) at fermentation onset to 14 g/100 g DM after the first day (Figure 2, purple line). After 3 days, sugar was almost completely depleted (0.8 g/100 g DM). Ethanol in the pulp rose to 28 g/100 g DM after 2 days (Figure 2, light green line), while acetic acid in the pulp reached 3.9 g/100 g DM (Figure 2, orange line). Ethanol in the cotyledons peaked at 1.0 g/100 g DM after 3 days and then declined (Figure 2, dark green line). The acetic acid concentration in the cotyledons rose substantially after day 2, remained at a level of ∼2 g/100 g DM between days 4 and 7, and then declined (Figure 2, red line). The temperature of the fermentation mass rose sharply from 32 to 47 °C after 2 days, remained at a level >45 °C for the next 3 days, and then decreased to 44 °C after 9 days (Figure 2, pink line). The pH of the pulp rose moderately from 3.8 to 4.6 after six fermentation days, then rapidly increased to 8.5 after 9 days (Figure 2, light blue line). The pH of the cotyledons first dropped from 6.8 to <5.0 after 4 days, further decreased from 4.9 to 4.4 between days 4 and 7, and then slightly increased again to reach 4.8 after 9 days (Figure 2, dark blue line). Well-fermented beans first appeared after 4 days of fermentation (Figure 2, yellow line). Their percentage rose sharply to 52% after 6 days. At this point, most of the pulp had been broken down, residual pulp had turned brown and separated from the beans. The well-fermented beans were purple-brown in color (cf. Figure 1), soft in texture and had a characteristic smell of acetic acid. After 10 days, the percentage of well-fermented beans rose to 100% and then dropped rapidly to zero, while overfermented beans increased to 100%. After 12 days, only a few dried pieces of dark brown to black pulp were left, and the cross sections revealed brown cotyledons with dark brown edges (cf. Figure 1). The texture of the beans was very soft, and the smell was strong and unpleasant with rotten, sweaty, and cheesy odors. Insect maggots were present in the fermentation mass, and mold was visible on the jute bag cover.

**Figure 2:**
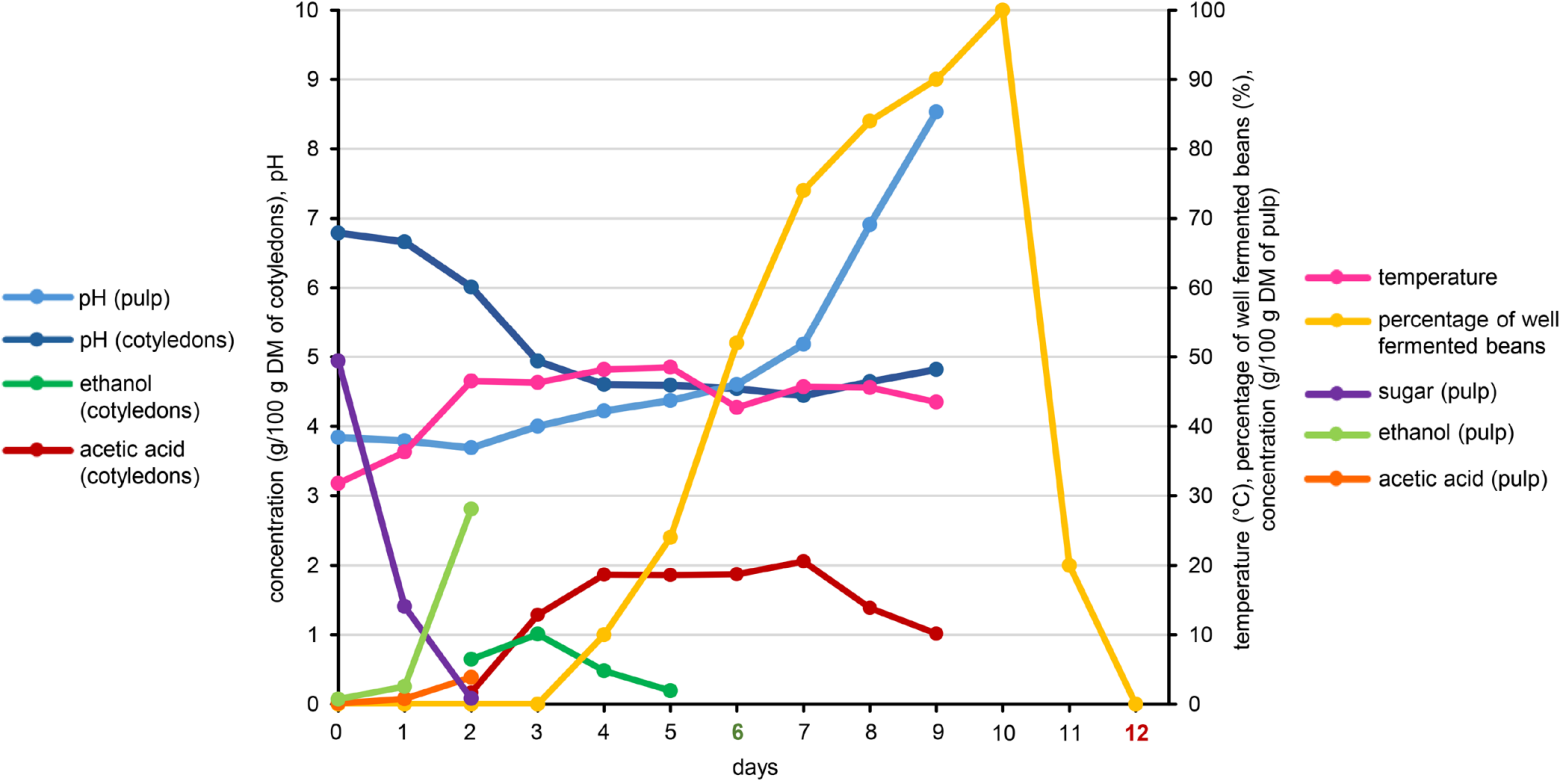
Parameters recorded on site during the cocoa fermentation process in Nicaragua: temperature of the fermentation mass, percentage of well-fermented beans, sugar concentration in the pulp, pH in cotyledons and pulp, ethanol and acetic acid concentrations in cotyledons and pulp.

The local protocol considered 70% of well-fermented beans as the threshold indicating the optimum fermentation end point. Therefore, when the percentage of well-fermented beans had reached 50% after 6 days, a follow-up cross-sectional analysis was performed 6 h later. This assessment finally revealed >70% well-fermented beans. Consequently, a representative sample was collected, dried, and considered as the optimally fermented reference sample for off-flavor compound quantitation. Its counterpart, the overfermented cocoa, was sampled after 12 days, when the percentage of overfermented beans had reached 100%.

Overall, the observations and data collected during the fermentation trial and their comparison with the literature confirmed that the two samples taken were representative of a well-fermented and an overfermented cocoa, respectively. In particular, proper cocoa fermentation was confirmed by a depletion of sugars during the first 3 days, followed by ethanol and acetic acid formation, subsequent penetration of acetic acid into the cotyledons associated with a pH drop to <5, temperatures exceeding 45 °C, and an acetic acid smell of the fermentation mass.^3,5,10,29,30^ On the other hand, overfermentation was indicated by a drop in the temperature of the fermentation mass, an increase in the pH of both cotyledons and pulp, blackening of the beans, and the development of an unpleasant smell.^9,20,21^ After drying, the well-fermented reference cocoa exhibited an aroma profile which an industry panel consisting of cocoa experts evaluated as characteristic.^31^ In contrast, the overfermented cocoa showed cheesy, sweaty, vomit-like, leathery, horse stable-like, and smoky odors.

### 3.2 Cocoa Samples from Nicaragua: Odorant Concentrations and OAVs

The concentrations of the selected off-flavor compounds in the cocoa nibs—which were essentially dried and crushed cotyledons—are listed in Table 1. The overfermented cocoa consistently showed higher concentrations than the reference cocoa in the phenolic compounds that have previously been identified as potential contributors to smoky off-flavors. The differences ranged from 1.2 to 20 times. In particular, smoky, sweet smelling 2-methoxyphenol and leathery, horse stable-like smelling 4-ethylphenol were substantially higher in the overfermented sample (224 and 48.4 μg/kg vs 75.2 and 2.19 μg/kg). By contrast, musty, earthy smelling geosmin was not higher in the overfermented sample (0.065 μg/kg) than in the reference (0.136 μg/kg). This, however, was the only exception among the compounds investigated, as the mushroom-like smelling oct-1-en-3-one and the five carboxylic acids were also present in higher concentrations in the overfermented cocoa than in the reference sample. The increase during overfermentation was rather small in acetic acid (20%); however, the sweaty, cheesy smelling compounds 2-methylpropanoic acid, butanoic acid, 2-methylbutanoic acid, and 3-methylbutanoic acid increased between 2-fold (3-methylbutanoic acid; from 65,100 μg/kg to 130,000 μg/kg) and almost 5-fold (2-methylpropanoic acid; from 35,000 μg/kg to 168,000 μg/kg) during overfermentation. Nevertheless, whether the higher concentrations in the overfermented samples have the potential to produce an off-flavor also depends on the OTCs of the individual compounds (cf. Table 1, column 3). For example, 4-methylphenol increased 2.7-fold during overfermentation; however, its final concentration (2.13 μg/kg) still remained below its OTC in deodorized cocoa butter (3.3 μg/kg) and therefore was unable to provoke an off-flavor.

**Table 1:** Concentrations of Off-Flavor Compounds in Dried Cocoa Nibs from Nicaragua: Reference Cocoa without Off-Flavor vs. Overfermented Cocoa

			concentration in nibs (μg/kg)^b^		
odorant	odor	OTC^a^	reference cocoa	overfermented cocoa	
3-methylphenol	smoky, phenolic	19	2.06	4.47	
4-methylphenol	phenolic, horse stable	3.3	0.799	2.13	
3-ethylphenol	smoky, phenolic	2.2	1.36	2.08	
4-ethylphenol	leathery, horse stable, smoky, phenolic	23	2.19	48.4	
2-methoxyphenol	smoky, sweet	1.8	75.2	224	
2,6-dimethoxyphenol	smoky, sweet, clove	83	15.9	19.0	
geosmin	musty, earthy, beetroot	1.6	0.136	0.0653	
oct-1-en-3-one	mushroom	0.93	0.241	0.634	
acetic acid	vinegar	400	2,420,000	2,900,000	
2-methylpropanoic acid	sweaty, cheesy	850	35,000	168,000	
butanoic acid	vomit, sweaty, rancid	52	883	3410	
2-methylbutanoic acid	sweaty, cheesy	130	11,900	33,400	
3-methylbutanoic acid	sweaty, cheesy	14	65,100	130,000	

a Odor threshold concentration in deodorized cocoa butter; OTCs of 3- and 4-methylphenol,^13^ 3- and 4-ethylphenol,^13^ 2-methoxyphenol,^13^ 2,6-dimethoxyphenol,^14^ and geosmin,^32^ were taken from the references cited; OTCs of oct-1-en-3-one, acetic acid, 2-methylpropanoic acid, butanoic acid, 2- and 3-methylbutanoic acid were determined within the current study according to a previously published approach.^13^

b Mean of triplicates; individual values and standard deviations are available in the Supporting Information file, Tables S3–S4.

To better assess and visualize the aroma potency of the individual odorants at the different determined concentration levels, OAVs were calculated by dividing the concentrations in the cocoa samples (cf. Table 1, columns 4 and 5) by the OTCs in deodorized cocoa butter (cf. Table 1, column 3) and the data were depicted in a bar chart (Figure 3). In this logarithmic chart, bars below the red horizontal line indicate OAVs < 1, i.e., concentrations below the respective OTC, and bars extending beyond the red line indicate OAVs > 1, i.e., concentrations above the OTC. OAVs below 0.1 are not depicted.

**Figure 3:**
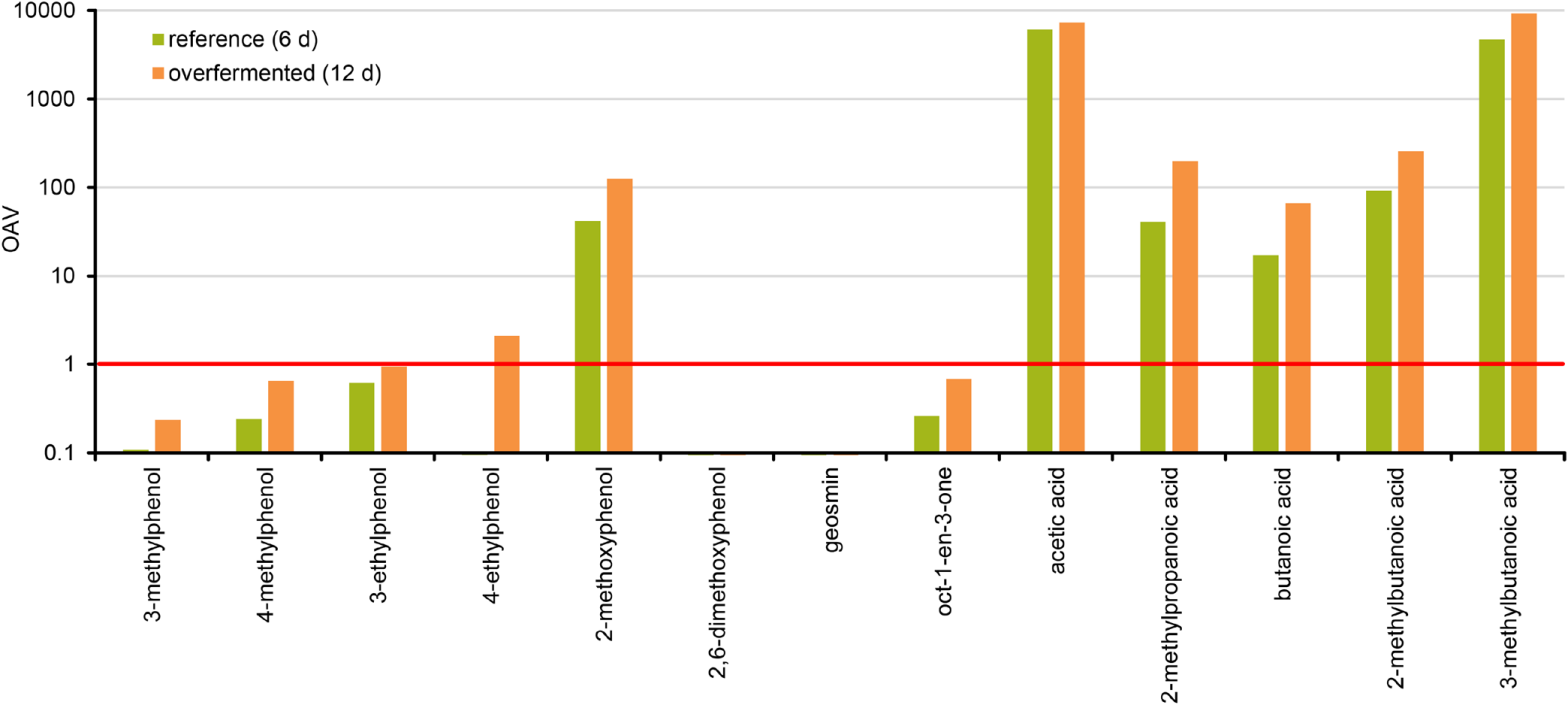
OAVs of off-flavor compounds in dried cocoa nibs from Nicaragua: reference cocoa without off-flavor vs overfermented cocoa.

Among the smoky smelling phenols, 2-methoxyphenol was the only compound that already exceeded its OTC in the reference sample. Despite an OAV of 42, no smoky off-flavor was detected in the reference sample. This was consistent with previous studies^13,17,33^ and also with the recently proposed maximum tolerable concentration of 70 μg/kg for 2-methoxyphenol in fermented cocoa,^13^ which corresponds to an OAV of 39. In the overfermented cocoa, however, the OAV of 2-methoxyphenol was 130 and thus above the previously suggested maximum tolerable level. In addition, leathery, horse stable-like smelling 4-ethylphenol, which was present in the reference sample in a concentration well below its odor threshold (OAV < 0.1), showed an OAV of 2.1 in the overfermented sample, while neither 3-methylphenol, nor 4-methylphenol, 3-ethylphenol, and 2,6-dimethoxyphenol exceeded their OTCs in the overfermented cocoa. The same applied to geosmin and oct-1-en-3-one.

By contrast, all of the carboxylic acids analyzed already exhibited high OAVs in the reference cocoa, ranging from 17 (butanoic acid) to 6100 (acetic acid). However, it must be taken into account that acidic compounds are generally overestimated when OAVs in cocoa are calculated based on OTCs in cocoa butter. Nonetheless, cocoa butter is generally considered the most appropriate matrix for OTC determinations and OAV calculations of cocoa odorants. It is particularly well suited for nondissociable lipophilic compounds. However, acidic compounds when dissolved in pure cocoa butter without the presence of an aqueous phase, remain in an undissociated state. In contrast, in the cocoa matrix, acidic compounds are partially deprotonated. This explains their overestimation when using OAVs based on OTCs in cocoa butter.^34^

During overfermentation, the OAVs of all carboxylic acids analyzed increased, with the greatest increase being found for sweaty, cheesy smelling 2-methylpropanoic acid (5-fold; from 41 to 200), followed by vomit-like, sweaty smelling butanoic acid (4-fold; from 17 to 66), sweaty, cheesy smelling 2-methylbutanoic acid (3-fold; from 92 to 257) and sweaty, cheesy smelling 3-methylbutanoic acid (2-fold; from 4700 to 9300).

In summary, the OAV data suggested that particularly smoky, sweet smelling 2-methoxyphenol, leathery, horse stable-like smelling 4-ethylphenol, sweaty, cheesy smelling 2-methylpropanoic acid, vomit-like, sweaty smelling butanoic acid, and sweaty, cheesy smelling 2- and 3-methylbutanoic acid contributed to the cheesy, sweaty, vomit-like, leathery, horse stable-like, and smoky smell of the overfermented sample.

Since the data obtained from the cocoa samples from Nicaragua only provided a single snapshot of overfermentation, our aim was to conduct a second fermentation trial on a cocoa farm in another country, using the clone mix and the fermentation parameters typical of that second location.

### 3.3 Cocoa Samples from Costa Rica: Fermentation Data

A major difference between the parameters in Nicaragua and Costa Rica was the batch size. Whereas in Nicaragua the fermentation started with 500 kg of wet mass, a smaller batch size of only 50 kg wet mass per box was employed in Costa Rica. Figure 4 shows the pods before opening, as well as the fermentation mass and the corresponding cross-sectional images of the beans at fermentation onset, after 6 days, and after 9 days of fermentation. The pods were ripe and showed no signs of disease. At the onset of fermentation, the whitish pulp adhered tightly to the firm beans. The beans had a homogeneous, dark purple color. The dark color corresponded to the local clone mix, which had a substantially higher polyphenol content than the clone mix used in Nicaragua (cf. Figure 1).

**Figure 4:**
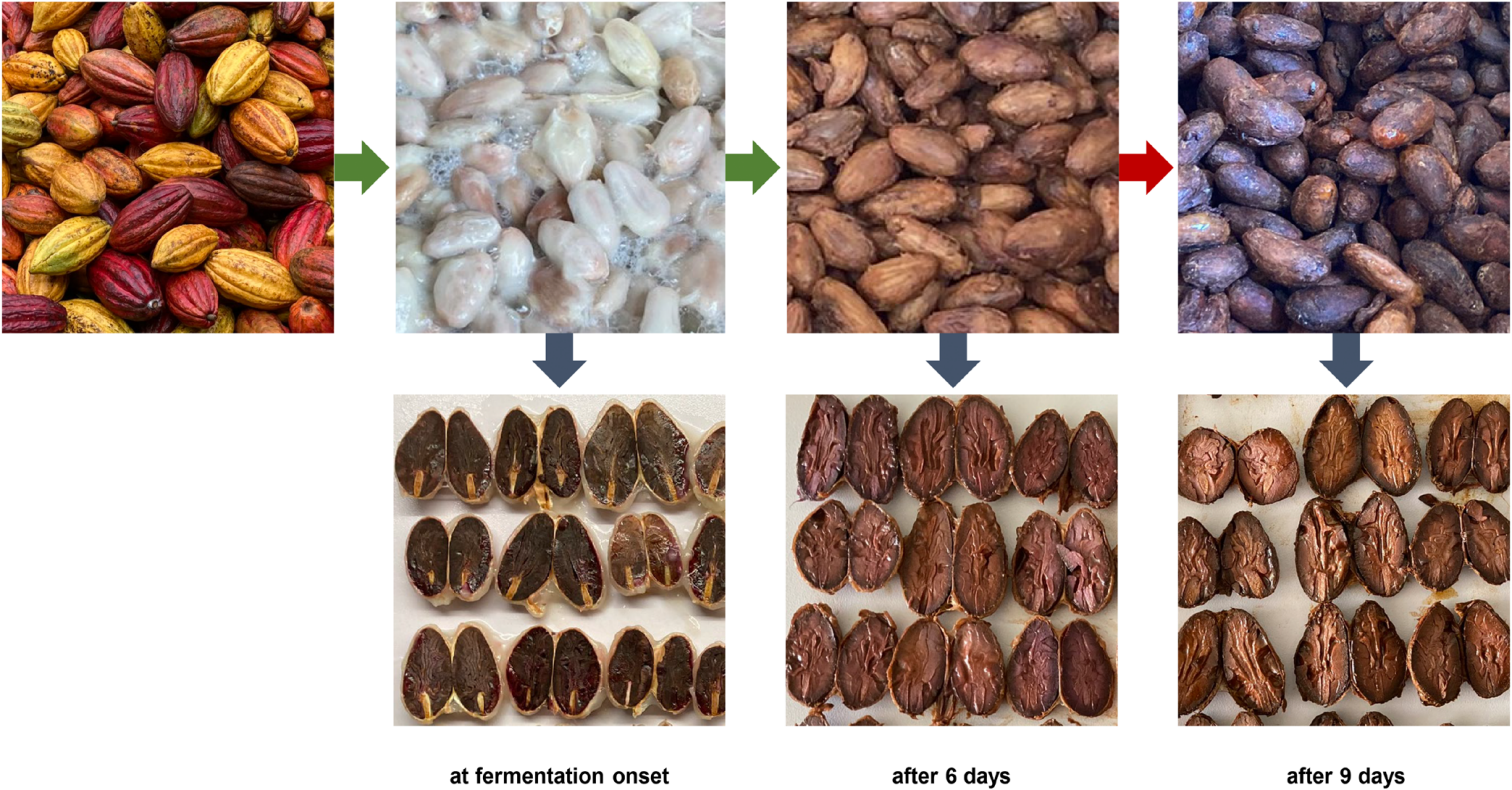
Cocoa fermentation in Costa Rica: cocoa pods before opening, fermentation mass and cross-sectional images on day 0, day 6 (reference sample), and day 9 (overfermented sample).

Figure 5 depicts the changes in physical and chemical key data during fermentation in Costa Rica. The parameters measured were the same as those in Nicaragua. Detailed data is available in the Supporting Information file, Table S6. During the first fermentation phase, the sugar content of the pulp decreased rapidly from an initial 75 g/100 g DM to 1.8 g/100 g DM after 2 days (Figure 5, purple line). Ethanol in the pulp peaked at 48 g/100 g DM after 2 days, whereas acetic acid in the pulp increased from 29 g/100 g DM at the start to 66 g/100 g DM after 3 days (Figure 5, light green and orange lines). In the cotyledons, the maximum ethanol concentration of 1.4 g/100 g DM was reached after 3 days and then decreased substantially until day 5 (Figure 5, dark green line). Acetic acid in the cotyledons peaked at 9.5 g/100 g DM after 4 days and then declined to 5.0 g/100 g DM by day 9 (Figure 5, red line). The temperature of the fermentation mass rose from 25 °C to ∼45 °C during the first 4 days of fermentation, remained at this level for two more days, and then dropped to 30 °C at days 8 and 9 (Figure 5, pink line). The pH of the pulp initially increased very slowly from 3.8 at the start to 4.5 after 6 days, and then quite rapidly to 8.6 after 9 days (Figure 5, light blue line). The pH of the cotyledons first decreased from 6.6 to below 5 after 4 days, remained at this level for two more days and then rose again to reach 6.2 after 9 days (Figure 5, dark blue line). Cross-sectional analysis revealed no well-fermented beans before day 5 of fermentation (Figure 5, yellow line). After day 5, their percentage rose sharply, reaching 86% after 6 days. At this stage, most of the pulp had decomposed, leaving only brown remnants loosely dispersed among the beans. The cross sections revealed purple-brown and soft beans that emitted a characteristic acetic acid odor (cf. Figure 4). After 7 days of fermentation, the percentage of well-fermented beans reached 96%, while 4% were already classified as overfermented. Finally, by day 9, well-fermented beans had declined to 8%, while the percentage of overfermented beans was 92%. At this stage, the pulp residues were brown to black in color and the fermentation mass emitted an unpleasant off-flavor with rotten, sweaty, and cheesy notes. The beans were soft, brown in the center, and some had thin dark brown edges (cf. Figure 4).

**Figure 5:**
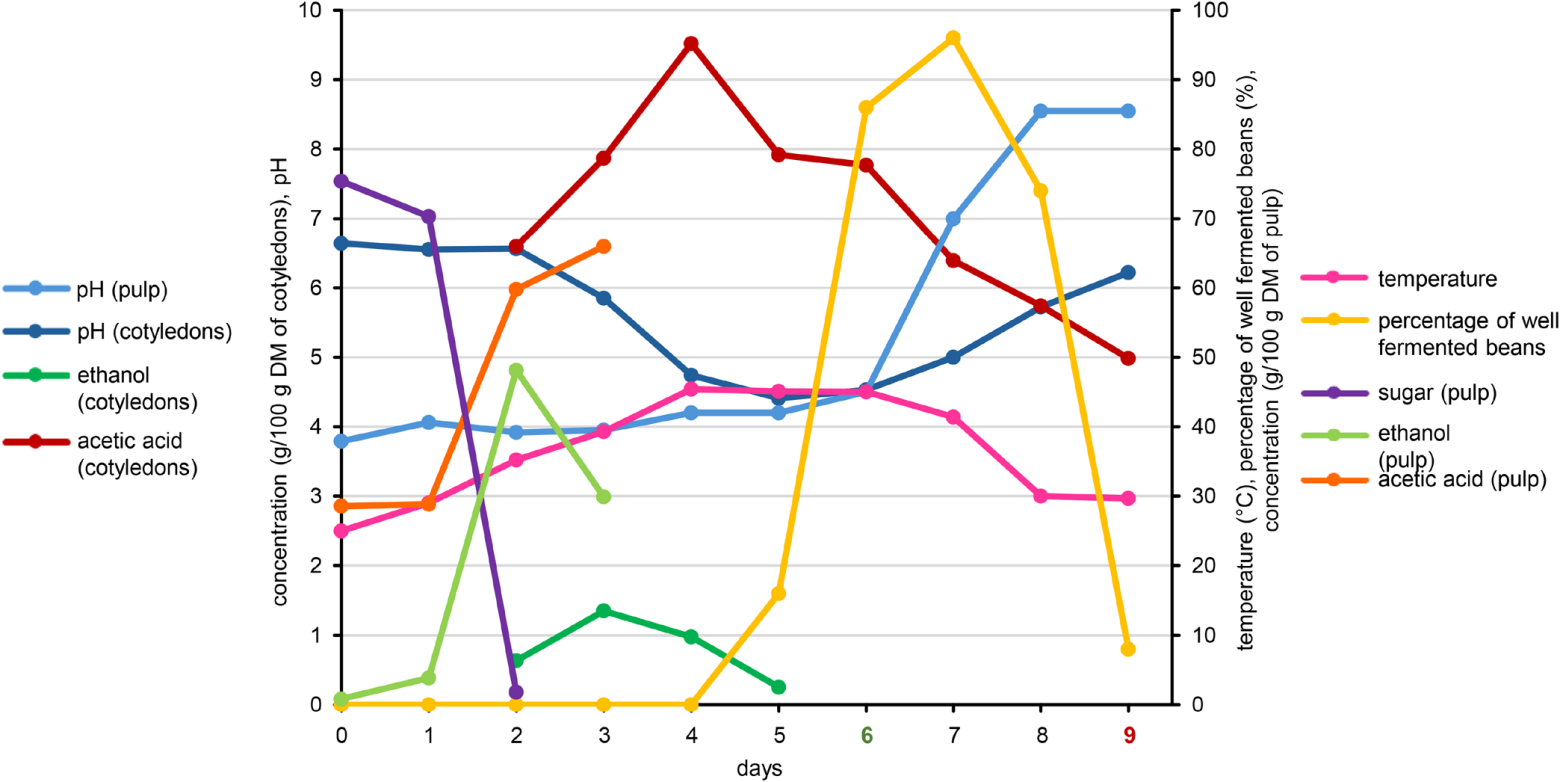
Parameters recorded on site during the cocoa fermentation process in Costa Rica: temperature of the fermentation mass, percentage of well-fermented beans, sugar concentration in the pulp, pH in cotyledons and pulp, ethanol and acetic acid concentrations in cotyledons and pulp.

As in Nicaragua, the fermentation end point corresponded to ∼70% well-fermented beans. Accordingly, a sample was taken after 6 days of fermentation, dried, and used as the optimally fermented reference for the quantitation of the off-flavor compounds. The corresponding overfermented cocoa was sampled after 9 days, when 92% of the beans were overfermented. As in Nicaragua, all observations confirmed the two samples taken to be representative of a well-fermented cocoa and an overfermented cocoa, respectively. The fact that the stage of overfermentation was reached after only 9 days in Costa Rica, whereas it took 12 days in Nicaragua, could be related to different weather conditions and different batch sizes. The weather in Costa Rica was consistently dry and warm, while in Nicaragua there had been regular rainfall and lower temperatures. On the other hand, the smaller fermentation batch size in Costa Rica may have promoted overfermentation through increased aeration.^20^ After drying, the well-fermented reference cocoa showed an aroma profile which an industry panel consisting of cocoa experts evaluated as typical of fermented cocoa,^31^ whereas the overfermented cocoa smelled of cheese, sweat, vomit, leather, horse stable, and smoke.

### 3.4 Cocoa Samples from Costa Rica: Odorant Concentrations and OAVs

Applying the same analytical procedures to the same selection of compounds as for the samples from Nicaragua resulted in the data summarized in Table 2. Among the phenolic compounds, 2-methoxyphenol and 4-ethylphenol again showed the greatest differences between the reference sample and the overfermented sample. During overfermentation, 2-methoxyphenol increased 10-fold (from 100 to 1000 μg/kg) and 4-ethylphenol 28-fold (from 1.62 to 45.1 μg/kg), while the other phenols showed only moderate changes. Geosmin increased 1.7-fold (from 0.047 to 0.079 μg/kg) and oct-1-ene-3-one 3.2-fold (from 0.738 to 2.34 μg/kg). In contrast to the samples from Nicaragua, the carboxylic acid concentrations were lower in Costa Rica, except for butanoic acid and did not increase consistently. Butanoic acid increased even more during overfermentation in Costa Rica (6-fold) than in Nicaragua (4-fold).

**Table 2:** Concentrations of Off-Flavor Compounds in Dried Cocoa Nibs from Costa Rica: Reference Cocoa without Off-Flavor vs. Overfermented Cocoa

			concentration in nibs (μg/kg)^b^	
odorant	odor	OTC^a^	reference cocoa	overfermented cocoa
3-methylphenol	smoky, phenolic	19	4.75	6.07
4-methylphenol	phenolic, horse stable	3.3	3.32	2.94
3-ethylphenol	smoky, phenolic	2.2	1.12	2.25
4-ethylphenol	leathery, horse stable, smoky, phenolic	23	1.62	45.1
2-methoxyphenol	smoky, sweet	1.8	100	1000
2,6-dimethoxyphenol	smoky, sweet, clove	83	6.51	8.68
geosmin	musty, earthy, beetroot	1.6	0.111	0.0794
oct-1-en-3-one	mushroom	0.93	0.738	2.34
acetic acid	vinegar	400	2,510,000	1,080,000
2-methylpropanoic acid	sweaty, cheesy	850	29,000	25,900
butanoic acid	vomit, sweaty, rancid	52	1070	6180
2-methylbutanoic acid	sweaty, cheesy	130	9790	7370
3-methylbutanoic acid	sweaty, cheesy	14	19,100	18,400

a Odor threshold concentration in deodorized cocoa butter; OTCs of 3- and 4-methylphenol,^13^ 3- and 4-ethylphenol,^13^ 2-methoxyphenol,^13^ 2,6-dimethoxyphenol,^14^ and geosmin,^32^ were taken from the references cited; OTCs of oct-1-en-3-one, acetic acid, 2-methylpropanoic acid, butanoic acid, 2- and 3-methylbutanoic acid were determined within the current study according to a previously published approach.^13^

b Mean of triplicates; individual values and standard deviations are available in the Supporting Information file, Tables S7–S8.

OAV calculations (Figure 6) confirmed the roles of 2-methoxyphenol and 4-ethylphenol for a potential leathery, horse stable-like and smoky off-flavor developed during overfermentation. With an OAV of 56 (Nicaragua: 42), 2-methoxyphenol was once again above its OTC even in the reference sample, albeit once more below the recently proposed maximum tolerable concentration of 70 μg/kg.^13^ After overfermentation in Costa Rica, 2-methoxyphenol reached an OAV as high as 560 (Nicaragua: 130). Like in Nicaragua, 4-ethylphenol increased during overfermentation from an olfactorily negligible amount (OAV < 0.1) to an amount with comparable odor activity (OAV 2.0 vs 2.1 in Nicaragua). Thus, in Costa Rica, just as in Nicaragua, 2-methoxyphenol, with its smoky, sweet odor, and 4-ethylphenol, with its leathery, horse stable-like odor were the only phenols that exceeded their OTCs in the overfermented sample, while 3-methylphenol, 4-methylphenol, 3-ethylphenol, and 2,6-dimethoxyphenol remained below an OAV of 1.

**Figure 6:**
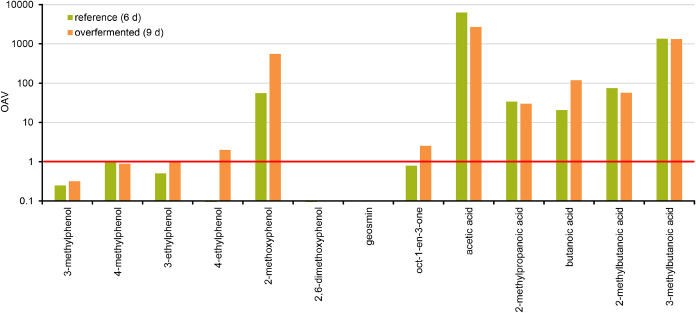
OAVs of off-flavor compounds in dried cocoa nibs from Costa Rica: reference cocoa without off-flavor vs overfermented cocoa.

While geosmin again showed negligible OAVs in both the reference and the overfermented sample, oct-1-en-3-one exceeded its OTC during overfermentation in Costa Rica, increasing from OAV 0.07 to 2.0. Thus, oct-1-en-3-one may contribute to a mushroom-like off-flavor that has occasionally been reported in fermented cocoa, which in turn could be an indication of overfermentation. As in Nicaragua, the OAV of vomit-like, sweaty smelling butanoic acid increased during overfermentation (6-fold from 21 to 120). In contrast to Nicaragua, however, the OAVs of the other carboxylic acids with cheesy odors, including 2-methylpropanoic acid, and 2- and 3-methylbutanoic acid, did not increase during overfermentation.

In summary, the OAV data suggested that smoky, sweet smelling 2-methoxyphenol, leathery, horse stable-like smelling 4-ethylphenol, and vomit-like, sweaty smelling butanoic acid contributed to the unpleasant off-flavor of the overfermented sample from Costa Rica.

### 3.5 Further Discussion and Perspectives

Despite the differences in the conditions under which the two overfermentation experiments were conducted in Nicaragua and Costa Rica, for example in terms of starting material, batch size, and local microflora, clear similarities emerged in the formation of the potential off-flavor compounds investigated. The most obvious parallel was found in the phenolic compounds. In both locations, 2-methoxyphenol and 4-ethylphenol increased substantially, exceeding their OTCs, during overfermentation. Although the OAVs of 4-ethylphenol were much lower than the OAVs of 2-methoxyphenol, 4-ethylphenol may have a substantial impact on the off-flavor, considering that its smoky odor is accompanied by harsh leathery, horse stable-like and phenolic nuances, whereas the smoky odor of 2-methoxyphenol is less harsh and sweeter.^14^ Moreover, the concentrations of 2-methoxyphenol and 4-ethylphenol in the two overfermented samples were in a similar range as previously reported in two samples of fermented cocoa with authentic wood smoke contact during drying in the origin.^14^ To the best of our knowledge, this is the first evidence that smoky smelling phenols can be formed in similarly high quantities during the overfermentation of cocoa as are found in cocoa with wood smoke contact during drying.

However, this only applied to 2-methoxyphenol and 4-ethylphenol. All other quantitated phenols, i.e., 3-methylphenol, 4-methylphenol, 3-ethylphenol, and 2,6-dimethoxyphenol, remained below their respective OTCs in both overfermented samples, whereas in the cocoa samples exposed to authentic wood smoke in the origin, all of them showed OAVs > 1, particularly 2,6-dimethoxyphenol, which was suggested as a marker compound for wood smoke contamination.^14^ Notwithstanding the small data set, the results suggest that in cocoa samples with a smoky off-flavor, the quantitative spectrum of odor-active phenols can provide information about whether the off-flavor is due to wood smoke contact or overfermentation. This can help clarify whether exposure to wood smoke or overfermentation plays a bigger role in the development of smoky off-flavors in cocoa.

While the odor-active phenols in wood smoke originate from the pyrolysis of lignin, the formation of 2-methoxyphenol and 4-ethylphenol during overfermentation is far less clear, although microbial formation is most likely. Phenolic acids such as vanillic acid, ferulic acid, and *p*-coumaric acid, potential precursors of 2-methoxyphenol and 4-ethylphenol, have been characterized in cocoa pulp and husks.^35–40^ Decarboxylation of vanillic acid formed from ferulic acid or other phenolic acids^41^ can lead to 2-methoxyphenol. Phenolic acid decarboxylase (PAD)-like enzymes, capable of metabolizing vanillic acid, have been detected in different *Bacillus* strains,^42,43^ including *Bacillus subtilis*,^44,45^ a bacterium that has already been considered responsible for the formation of off-flavors in overfermented cocoa.^46^ Nevertheless, while this metabolic pathway contributes to the spoilage of other foods,^44,47^ it has not yet been shown to play a role in the overfermentation of cocoa.

A plausible route to 4-ethylphenol starts with the microbial decarboxylation of *p*-coumaric acid to 4-vinylphenol, which subsequently undergoes reduction. The first step is also catalyzed by PAD-like enzymes found in microorganisms such as *Bacillus* ^48^ and *Lactobacillus*,^49,50^ which typically grow in the later stages of cocoa fermentation. Reduction of 4-vinylphenol to 4-ethylphenol has been reported to occur in yeasts such as *Brettanomyces* ^51,52^ and some lactic acid bacteria.^53^ The formation of 4-ethylphenol during overfermentation may therefore require an interspecific metabolic interaction. Although the proposed pathways to 2-methoxyphenol and 4-ethylphenol have not been experimentally confirmed in cocoa, their feasibility is supported by the presence of the relevant substrates in cocoa and by the metabolic potential of the microbial community typically active during late-stage fermentation.

Another parallel in the results of the overfermentation experiments conducted in Nicaragua and Costa Rica was the increase in the vomit-like, sweaty smelling butanoic acid. In Nicaragua, this substance quadrupled from 883 to 3410 μg/kg during overfermentation, while in Costa Rica it increased 6-fold from 1070 to 6180 μg/kg. However, the other cheesy smelling compounds quantitated behaved differently during overfermentation in Nicaragua and Costa Rica. In Nicaragua, 2-methylpropanoic acid, 2-methylbutanoic acid, and 3-methylbutanoic acid also substantially increased, specifically 5-fold, 3-fold, and 2-fold, respectively. By contrast, these compounds did not increase during overfermentation in Costa Rica, which suggested that butanoic acid was crucial for the vomit-like, cheesy off-flavor in the overfermented sample from Costa Rica. In this situation, we gained access to another overfermented cocoa originating in Ecuador and the corresponding optimally fermented reference. However, on-site analyses data were not available for this sample. Interestingly, this cocoa also showed an increase in butanoic acid during overfermentation, but only from 1210 to 1550 μg/kg, corresponding to a plus of 28% (Supporting Information file, Tables S10–S12). In Ecuador, the OAVs of all cheesy smelling carboxylic acids substantially increased, although on a lower level compared to Nicaragua. In both cases, this increase was mainly due to 3-methylbutanoic acid, whose OAV increased from 830 to 2100 in Ecuador and from 4700 to 9300 in Nicaragua.

In summary, the data suggested that overfermentation can be associated with an increase of different cheesy smelling carboxylic acids such as butanoic acid and 3-methylbutanoic acid, although a general pattern could not be identified. This has been assumed before,^9,19^ but an increase in these compounds has also been reported to already occur in the final stages of an optimal fermentation process.^19,54–57^ Lopez and Quesnel attributed the increase in the cheesy smelling carboxylic acids to excessive aeration, caused by a low pulp content in the fermentation mass.^20^ Indeed, also slits in the fermentation box fostering oxygen access already led to blackened beans and elevated levels of the cheesy smelling carboxylic acids after only 5 days of fermentation (cf. Supporting Information file, Tables S13–S15). The formation of cheesy smelling carboxylic acids is mainly attributed to the dehydrogenase activity of *Bacillus* species and aerotolerant lactic acid bacteria, which are frequently detected in the late aerobic stages of cocoa fermentation, and are able to convert amino acids into the corresponding acids.^9,20,30,46^

In both Nicaragua and Costa Rica, geosmin formation did not play a crucial role as concentrations remained well below the OTC. In general, geosmin is a metabolite produced by certain actinomycetes, cyanobacteria, myxobacteria, and fungi found in aquatic and terrestrial environments.^58–63^ While such bacteria have not been detected in cocoa fermentation, geosmin-producing fungi are known spoilage organisms in cocoa postharvest processing.^46,60^ Moreover, moldy-musty off-flavors in cocoa are often associated with visible mold growth,^15^ although it is known that only a minority of mold species can produce geosmin. Geosmin formation during cocoa fermentation might therefore depend on the presence of very specific fungi; however, their identity is yet unknown.

Mushroom-like smelling oct-1-en-3-one showed higher concentrations in the overfermented samples than in the references at both locations. However, only in Costa Rica the concentration in the overfermented sample surpassed the OTC. Oct-1-en-3-one is another fungal metabolite. It is formed from unsaturated fatty acids under aerobic conditions.^64^ In cocoa fermentations, both the substrates and oct-1-en-3-one-producing fungi have been detected.^30,46,65,66^

In conclusion, the results of this study suggest that cocoa overfermentation can lead to smoky, leathery and horse stable-like off-flavors caused by 2-methoxyphenol and 4-ethylphenol as well as to cheesy, vomit-like off-flavors caused by short-chain carboxylic acids such as 2-methylpropanoic acid, butanoic acid, 2-methylbutanoic acid, and 3-methylbutanoic acid. However, further studies using sensory methods are needed to better understand at what concentrations these compounds are actually perceived as off-flavors and which microbial communities promote their formation. In addition to the new findings on the formation of off-flavor compounds during cocoa overfermentation, the on-site analyses performed on the cocoa farms yielded another interesting result: the pH of the pulp, which rises during fermentation, exceeded a value of 4.5 at both locations when the optimal fermentation time was reached. Thus, measuring the pH of the pulp, by simply dipping a pH strip into the fermentation mass could potentially be a simple tool to detect the onset of overfermentation.

## Supplementary Material



## Notes

Additional information on materials and methods used during the fermentation trials in Nicaragua and Costa Rica; additional information on quantitation assays; additional information on GC–MS instruments; stable isotopically substituted internal standards, quantifier ions, and calibration lines used in the GC–MS quantitations; detailed data on the fermentation processes in Nicaragua and Costa Rica; individual concentrations used for mean calculations and standard deviations; OAV data depicted in Figures 3 and 6; data obtained from additional fermentation trials in Costa Rica and Ecuador (PDF)

## Abbreviations

CI: chemical ionization
DM: dry matter
GC: gas chromatography
GC–MS: gas chromatography–mass spectrometry
GC–GC–HRMS: gas chromatography–gas chromatography–high resolution mass spectrometry
OAV: odor activity value
OTC: odor threshold concentration
PAD: phenolic acid decarboxylase
