# Supplementary material for: Off-Flavor Compounds in Fermented Cocoa: Impact of Overfermentation
Source: J Agric Food Chem. 2026 Jul 14;74(29):23069–80. doi: 10.1021/acs.jafc.6c05685 (PMC13426307; doi:10.1021/acs.jafc.6c05685)
Supplement: Supplementary file 1 [file jf6c05685_si_001.pdf]

Supporting Information

Off-Flavor Compounds in Fermented Cocoa: Impact of Overfermentation

Franziska Krause[1,2] and Martin Steinhaus[2,1,*]

[1] Technical University of Munich, TUM School of Natural Sciences, Department of Chemistry, Lichtenbergstraße 4, 85748 Garching, Germany

[2] Leibniz Institute for Food Systems Biology at the Technical University of Munich (Leibniz-LSB@TUM), Lise-Meitner-Straße 34, 85354 Freising, Germany

*E-mail: m.steinhaus.leibniz-lsb@tum.de

Overview

Additional Information

Fermentation Trials in Nicaragua

Fermentation Trials in Costa Rica

Quantitation Assays

GC–MS Instrument

Heart-Cut GC–GC–HRMS Instrument

Additional Tables

Table S1. Stable Isotopically Substituted Internal Standards, Quantifier Ions, and Calibration Lines Used in the GC–MS Quantitations

Table S2. Cocoa Samples from Nicaragua: Fermentation Data

Table S3. Concentrations of Off-Flavor Compounds in Dried Cocoa Nibs from Nicaragua: Reference Cocoa without Off-Flavor

Table S4. Concentrations of Off-Flavor Compounds in Dried Cocoa Nibs from Nicaragua: Overfermented Cocoa

Table S5. OAVs of Off-Flavor Compounds in Dried Cocoa Nibs from Nicaragua: Reference Cocoa without Off-Flavor vs. Overfermented Cocoa

Table S6. Cocoa Samples from Costa Rica: Fermentation Data

Table S7. Concentrations of Off-Flavor Compounds in Dried Cocoa Nibs from Costa Rica: Reference Cocoa without Off-Flavor

Table S8. Concentrations of Off-Flavor Compounds in Dried Cocoa Nibs from Costa Rica: Overfermented Cocoa

Table S9. OAVs of Off-Flavor Compounds in Dried Cocoa Nibs from Costa Rica: Reference Cocoa without Off-Flavor vs. Overfermented Cocoa

Table S10. Concentrations and OAVs of Off-Flavor Compounds in Dried Cocoa Nibs from Ecuador: Reference Cocoa without Off-Flavor vs. Overfermented Cocoa

Table S11. Concentrations of Off-Flavor Compounds in Dried Nibs of the Reference Cocoa without Off-Flavor from Ecuador

Table S12. Concentrations of Off-Flavor Compounds in Dried Nibs of the Overfermented Cocoa from Ecuador

Table S13. Concentrations and OAVs of Off-Flavor Compounds in Dried Cocoa Nibs from Costa Rica: Reference Cocoa without Off-Flavor vs. Cocoa Fermented in a Box with Side Slits

Table S14. Concentrations of Off-Flavor Compounds in Dried Nibs of the Second Reference Cocoa without Off-Flavor from Costa Rica

Table S15. Concentrations of Off-Flavor Compounds in Dried Nibs of the Cocoa Sample Fermented in a Box with Side Slits from Costa Rica

Additional Information

Fermentation Trials in Nicaragua

Each day, fifty beans were cut lengthwise and individually classified by the local plantation staff as unfermented, semi-fermented, well-fermented, or overfermented. Subsequently, one half of each bean was separated, the seed coats were removed by hand, and the cotyledons were ground. The pH values were determined with a calibrated pH meter (Milwaukee; Brookfield, Wisconsin). The pH of the pulp was measured after stirring a sample (20 g) of whole beans with adhering pulp and distilled water (10 mL) for 10 sec. The pH of the cotyledons was measured after mixing a sample (5 g) of ground cotyledons with distilled water (45 mL) and waiting for 5 min. The water content of pulp (1 g) and cotyledons (ground; 5 g) was determined using a halogen moisture analyzer (Ohaus; Nänikon, Switzerland). Sugar, ethanol, and acetic acid concentrations were determined enzymatically after Carrez clarification using Yellow Line Roche Diagnostics test kits from R-Biopharm (Darmstadt, Germany) in combination with a photometer 680 operated at 340 nm (Robert Riele; Berlin, Germany). For Carrez clarification, according to the manufacturer's instructions, 100 mL of distilled water and Carrez solutions 1 and 2 (3 ml each) were added to samples (10 g) of pulp or ground cotyledons. The pH was adjusted to 7 using aqueous NaOH (1 mol/L), the volume was brought up to 250 mL with distilled water, and the mixture was filtered through a folded paper filter.

Based on the results of the on-site analyses, a reference sample (50 kg) and an overfermented sample (50 kg) were collected from the fermentation mass after 6 and 12 days, respectively. The samples were dried according to local practices with a layer thickness of 10–15 cm and with mixing every 2–3 h. After two days, the drying process was assisted by alternating flows of warm air at 60 °C and ambient air. When the moisture content was below 8%, the beans were vacuum-packed and shipped to Germany for the quantitation of the off-flavor compounds.

Fermentation Trials in Costa Rica

The sampling and the on-site analysis followed the same procedure as in Nicaragua; however, the pH meter was from Greisinger (Regenstauf, Germany). Furthermore, the moisture content determination was not automatized. Instead, pulp (1 g) or ground cotyledons (2 g) were dried in a pre-weighed weighing dish with a ground glass joint at 100 °C for 1 h, after which the glass was sealed, allowed to cool to room temperature, and re-weighed.

Based on the results of the on-site analyses, a reference sample (6 kg) and an overfermented sample (6 kg) were collected from the fermentation mass after 6 and 9 days, respectively, sun-dried according to local practices by spreading the beans in a single layer and mixing every 12 h. When the moisture content determined with a moisture meter HE 50 (Pfeuffer; Kitzingen, Germany) was below 8%, the beans were vacuum-packed and shipped to Germany for the quantitation of the off-flavor compounds.

Quantitation Assays

Fermented cocoa beans were first separated into a nibs and a husks fraction using a cocoa breaker and a winnower with a vibratory feeder (Commodity Processing Systems; Colchester, UK). Breaker and winnower were thoroughly cleaned after each sample to prevent carryover. The nibs fractions were further purified by manual sorting to obtain a 100% nibs sample. Each nibs fraction was separately ground to a fine powder (0.5–50 g) using a 6875 Freezer Mill (SPEX SamplePrep; Stanmore, UK). The powders were stirred with water (4–100 mL) for 10 minutes. Dichloromethane (40–400 mL) was added together with stable isotopically substituted odorants used as internal standards. The amount of internal standard varied between 0.01 and 550 µg,

depending on the expected target compound concentration and the amount of sample used for the workup. The mixtures were stirred overnight at ambient temperature, dried over anhydrous sodium sulfate, filtered, and nonvolatiles were removed by SAFE at 40 °C (cf. *Eur. Food Res. Technol.* **1999**, *209*, 237–241). Volatile isolates were concentrated to a final volume of 200 µL, first using a Vigreux column (50 × 1 cm) and then a microdestillation device (cf. In *Progress in Flavour Research*; Land, G. G.; Nursten, H. E., Eds.; Applied Science Publishers: London, UK, 1979; pp. 79−88.), and analyzed by GC–MS. Peak areas corresponding to the analytes and the internal standards were obtained from extracted ion chromatograms using characteristic quantifier ions. Odorant concentrations in the cocoa samples were finally calculated from the area counts, the amount of internal standard added, and the amount of the cocoa sample used for the workup with the help of a calibration line equation. Individual calibration line equations were obtained from odorant/standard mixtures with different concentration ratios (1:10, 1:5, 1:2, 1:1, 2:1, 5:1, and 10:1), which had been analyzed under identical conditions, followed by linear regression. Quantitation assays were carried out in triplicate.

GC–MS Instrument

A 7890B gas chromatograph (Agilent; Waldbronn, Germany) equipped with a Varian PAL autosampler (Varian; Darmstadt, Germany) and a cold on-column injector was used to analyze acetic acid, 2-methylpropanoic acid, and butanoic acid. Separation was achieved on a DB-FFAP column (30 m × 0.25 mm i.d., 0.25 µm film thickness; Agilent). Helium served as the carrier gas at a constant flow of 1.0 mL/min, and the injection volume was 1 µL. The oven temperature was initially held at 40 °C for 2 min, then increased at 6 °C/min to 230 °C, where it was held for 5 min. The GC was coupled to a Varian 220 ion trap mass spectrometer operated in the chemical ionization (CI) mode with methanol as the reagent gas and a scan range of *m*/*z* 60–100. Data acquisition and processing were performed using MS Workstation software, version 7.0.2 (Agilent).

Heart-Cut GC–GC–HRMS Instrument

The two-dimensional heart-cut GC–GC–high resolution mass spectrometry (HRMS) system consisted of two Trace 1310 gas chromatographs (Thermo Fisher Scientific; Waltham, MA, USA) connected with a Deans switch device (S+H Analytik; Mönchengladbach, Germany), and a high-resolution Q Exactive GC Orbitrap mass spectrometer (Thermo Fisher Scientific). The first GC was equipped with a TriPlus RSH autosampler, a programmed temperature vaporizing (PTV) injector, and a DB-FFAP column, 30 m × 0.32 mm i.d., 0.25 µm thickness (Agilent); an FID (250 °C base temperature) and a custom-made sniffing port served as monitor detectors. The carrier gas was helium at a constant flow of 1.0 mL/min. The injection volume was 1–2 µL. The initial oven temperature of 40 °C was held for 2 min and then increased to 230 °C by 6 °C/min. The final temperature was held for 5 min. The end of the column was connected to the Deans switch, which directed the column effluent time-programmed through uncoated but deactivated fused silica capillaries (0.25 mm i.d.) either to the monitor detectors or via a heated hose (250 °C) to a liquid nitrogen-cooled trap. The trap was connected to the column in the second GC, which was a DB-1701 column, a DB-FFAP column, (both 30 m × 0.25 mm, i.d., 0.25 µm film thickness), or a DB-5 column, (30 m × 0.25 mm, i.d., 1 µm film thickness; Agilent). The DB-1701 column was used for the analysis of 2-methoxyphenol, 2,6-dimethoxyphenol, geosmin, and oct-1-en-3-one, the DB-FFAP column was used for the analysis of 3- and 4-methylphenol as well as 3- and 4-ethylphenol, and the DB-5 column was used for the analysis of 2- and 3-methylbutanoic acid. The initial temperature of the second oven was 40 °C, held for 2 min, and then increased to 230 °C by 6 °C/min. The final temperature was held for 5 min. The end of the second GC column was connected to the mass spectrometer operated in high-resolution mode with positive CI, using isobutane as reagent gas and a scan range of *m*/*z* 90–200. Data evaluation was accomplished with the Xcalibur software (Thermo Fisher Scientific).

Additional Tables

Table S1. Stable Isotopically Substituted Internal Standards, Quantifier Ions, and Calibration Lines Used in the GC–MS Quantitations

odorant	internal standard	quantifier ions (*m*/*z*)		calibration line equation	R^2
		analyte	standard		
3-methylphenol	($^2H_7$)-4-methylphenol	109.0638	113.0899<br>114.0962<br>115.1025<br>116.1087<br>117.1150	y = 1.1586x − 0.3972[a]	0.974
4-methylphenol	($^2H_7$)-4-methylphenol	109.0638	113.0899<br>114.0962<br>115.1025<br>116.1087<br>117.1150	y = 1.1236x − 0.0979[a]	0.999
3-ethylphenol	($^2H_{2-3}$)-4-ethylphenol	123.0804	125.0930<br>126.0993	y = 0.8983x − 0.0532[b]	0.999
4-ethylphenol	($^2H_{2-3}$)-4-ethylphenol	123.0804	125.0930<br>126.0993	y = 0.8343x − 0.0095[b]	0.999
2-methoxyphenol	($^2H_3$)-2-methoxyphenol	125.0597	128.0785	y = 1.0704x − 0.0821[a]	0.999
2,6-dimethoxyphenol	($^2H_{5-8}$)-2,6-dimethoxyphenol	155.0703	157.0828<br>158.0891<br>159.0954<br>160.1017<br>161.1079<br>162.1142<br>163.1205	y = 1.9959x − 0.1323[a]	0.999
geosmin	($^2H_3$)-(–)-geosmin	165.1638	168.1826	y = 1.3150x − 0.0210[b]	0.999
oct-1-en-3-one	($^2H_{2-4}$)oct-1-en-3-one	127.1117	129.1243<br>130.1306<br>131.3684	y = 0.8631x − 0.0656[a]	0.998
acetic acid	($^2H_3$)acetic acid	61	64	y = 1.0947x − 0.0721[a]	0.995
2-methylpropanoic acid	($^2H_7$)-2-methylpropanoic acid	89	96	y = 0.9844x + 0.0198[b]	0.999
butanoic acid	($^2H_3$)butanoic acid	89	91	y = 0.9985x + 0.1332[a]	0.995
2-methylbutanoic acid	($^2H_9$)-2-methylbutanoic acid	103.0754	112.1319	y = 0.9668x − 0.0637[b]	0.996
3-methylbutanoic acid	($^2H_2$)-3-methylbutanoic acid	103.0754	105.0879	y = 0.9621x − 0.0817[a]	0.999

[a]y = peak area counts analyte / peak area counts standard; x = concentration analyte (µg/kg) / concentration standard (µg/kg). [b]y = peak area counts standard / peak area counts analyte; x = concentration standard (µg/kg) / concentration analyte (µg/kg).

Table S2. Cocoa Samples from Nicaragua: Fermentation Data

	day 0	day 1	day 2	day 3	day 4	day 5	day 6	day 7	day 8	day 9	day 10	day 11	day 12
temperature, center of the box[a] (°C)	31.8	36.3	46.5	46.3	48.2	48.5	42.7	45.7	45.6	43.5			
pH of pulp	3.8	3.8	3.7	4.0	4.2	4.4	4.6	5.2	6.9	8.5			
pH of nibs	6.8	6.7	6.0	4.9	4.6	4.6	4.5	4.4	4.6	4.8			
well fermented beans[b] (%)	0	0	0	0	10	24	52	74	84	90	100	20	0
acetic acid in pulp (g/100 g DM)	0.1	0.8	3.9										
acetic acid in nibs (g/100 g DM)			0.2	1.3	1.9	1.9	1.9	2.1	1.4	1.0			
ethanol in pulp (g/100 g DM)	0.7	2.6	28										
ethanol in nibs (g/100 g DM)			0.6	1.0	0.5	0.2							
sugar[c] in pulp (g/100 g DM)	49	14	0.8										

[a]Measured in the center of the fermentation box. [b]Percentage of well fermented beans as determined by cut tests carried out by experienced personnel on site. [c]Sum of glucose and sucrose.

Table S3. Concentrations of Off-Flavor Compounds in Dried Cocoa Nibs from Nicaragua: Reference Cocoa without Off-Flavor

odorant	concentration (µg/kg)			
	experiment 1	experiment 2	experiment 3	mean ± SD (CV)[a]
3-methylphenol	2.31	1.97	1.89	2.06 ± 0.18 (9%)
4-methylphenol	0.928	0.836	0.633	0.799 ± 0.123 (15%)
3-ethylphenol	1.13	1.60	1.35	1.36 ± 0.19 (14%)
4-ethylphenol	1.94	1.62	3.02	2.19 ± 0.60 (27%)
2-methoxyphenol	73.2	74.4	78.1	75.2 ± 2.1 (3%)
2,6-dimethoxyphenol	21.4	9.44	16.8	15.9 ± 4.9 (31%)
geosmin	0.092	0.0503	0.267	0.136 ± 0.094 (69%)
oct-1-en-3-one	0.272	0.249	0.203	0.241 ± 0.029 (12%)
acetic acid	2260000	2540000	2450000	2420000 ± 116000 (5%)
2-methylpropanoic acid	33000	35200	36700	35000 ± 1520 (4%)
butanoic acid	808	888	954	883 ± 60 (7%)
2-methylbutanoic acid	11600	10900	13300	11900 ± 1030 (9%)
3-methylbutanoic acid	64300	65300	65800	65100 ± 612 (1%)

[a]SD, standard deviation; CV, coefficient of variation.

Table S4. Concentrations of Off-Flavor Compounds in Dried Cocoa Nibs from Nicaragua: Overfermented Cocoa

odorant	concentration (µg/kg)			
	experiment 1	experiment 2	experiment 3	mean ± SD (CV)[a]
3-methylphenol	4.36	3.96	5.09	4.47 ± 0.47 (10%)
4-methylphenol	2.32	1.52	2.54	2.13 ± 0.44 (21%)
3-ethylphenol	2.23	0.737	3.27	2.08 ± 1.04 (50%)
4-ethylphenol	40.6	53.0	51.6	48.4 ± 5.6 (11%)
2-methoxyphenol	216	222	236	224 ± 9 (4%)
2,6-dimethoxyphenol	21.8	15.9	19.4	19.0 ± 2.4 (13%)
geosmin	0.0393	0.0864	0.0701	0.0653 ± 0.0195 (30%)
oct-1-en-3-one	0.681	0.598	0.623	0.634 ± 0.035 (5%)
acetic acid	3000000	2760000	2960000	2900000 ± 107000 (4%)
2-methylpropanoic acid	180000	163000	161000	168000 ± 839 (5%)
butanoic acid	3290	3620	3310	3410 ± 152 (4%)
2-methylbutanoic acid	34700	33300	32300	33400 ± 982 (3%)
3-methylbutanoic acid	135000	124000	132000	130000 ± 4800 (4%)

[a]SD, standard deviation; CV, coefficient of variation.

Table S5. OAVs of Off-Flavor Compounds in Dried Cocoa Nibs from Nicaragua: Reference Cocoa without Off-Flavor vs. Overfermented Cocoa

odorant	odor activity value (OAV)[a]	
	reference cocoa	overfermented cocoa
3-methylphenol	0.11	0.24
4-methylphenol	0.24	0.65
3-ethylphenol	0.62	0.95
4-ethylphenol	0.095	2.1
2-methoxyphenol	42	130
2,6-dimethoxyphenol	0.19	0.23
geosmin	0.085	0.041
oct-1-en-3-one	0.26	0.68
acetic acid	6100	7300
2-methylpropanoic acid	41	200
butanoic acid	17	66
2-methylbutanoic acid	92	260
3-methylbutanoic acid	4700	9300

[a]OAVs were calculated as mean concentration/OTC.

Table S6. Cocoa Samples from Costa Rica: Fermentation Data

	day 0	day 1	day 2	day 3	day 4	day 5	day 6	day 7	day 8	day 9
temperature, center of the box[a] (°C)	25.0	29.0	35.2	39.3	45.4	45.1	45.0	41.4	30.0	29.7
pH of pulp	3.8	4.1	3.9	4.0	4.2	4.2	4.5	7.0	8.6	8.6
pH of nibs	6.6	6.6	6.6	5.9	4.7	4.4	4.5	5.0	5.7	6.2
well fermented beans[b] (%)	0	0	0	0	0	16	86	96	74	8
acetic acid in pulp (g/100 g DM)	29	29	60	66						
acetic acid in nibs (g/100 g DM)			6.6	7.9	9.5	7.9	7.8	6.4	5.7	5.0
ethanol in pulp (g/100 g DM)	0.8	3.8	48	30						
ethanol in nibs(g/100 g DM)			0.6	1.3	1.0	0.3				
sugar[c] in pulp (g/100 g DM)	75	70	1.8							

[a]Measured in the center of the fermentation box. [b]Percentage of well fermented beans as determined by cut tests carried out by experienced personnel on site. [c]Sum of glucose and sucrose.

Table S7. Concentrations of Off-Flavor Compounds in Dried Cocoa Nibs from Costa Rica: Reference Cocoa without Off-Flavor

odorant	concentration (µg/kg)			
	experiment 1	experiment 2	experiment 3	mean ± SD (CV)[a]
3-methylphenol	5.08	4.49	4.69	4.75 ± 0.24 (5%)
4-methylphenol	4.67	2.46	2.82	3.32 ± 0.97 (29%)
3-ethylphenol	0.496	0.765	2.11	1.12 ± 0.70 (63%)
4-ethylphenol	0.957	1.48	2.41	1.62 ± 0.60 (37%)
2-methoxyphenol	104	97.9	98.3	100 ± 3 (3%)
2,6-dimethoxyphenol	7.11	7.47	4.95	6.51 ± 1.11 (17%)
geosmin	0.240	0.0451	0.0489	0.111 ± 0.091 (82%)
oct-1-en-3-one	0.585	0.751	0.878	0.738 ± 0.120 (16%)
acetic acid	2500000	2510000	2520000	2510000 ± 8130 (0%)
2-methylpropanoic acid	28800	29100	28900	29000 ± 143 (0%)
butanoic acid	1060	1090	1070	1070 ± 13 (1%)
2-methylbutanoic acid	9450	9740	10200	9790 ± 304 (3%)
3-methylbutanoic acid	19000	18800	19400	19100 ± 228 (1%)

[a]SD, standard deviation; CV, coefficient of variation.

Table S8. Concentrations of Off-Flavor Compounds in Dried Cocoa Nibs from Costa Rica: Overfermented Cocoa

odorant	concentration (µg/kg)			
	experiment 1	experiment 2	experiment 3	mean ± SD (CV)[a]
3-methylphenol	6.09	6.26	5.87	6.07 ± 0.16 (3%)
4-methylphenol	3.10	2.87	2.85	2.94 ± 0.11 (4%)
3-ethylphenol	2.53	1.44	2.79	2.25 ± 0.58 (26%)
4-ethylphenol	46.7	44.1	44.5	45.1 ± 1.2 (3%)
2-methoxyphenol	1020	1020	956	1000 ± 31 (3%)
2,6-dimethoxyphenol	9.22	6.33	10.5	8.68 ± 1.74 (20%)
geosmin	0.0761	0.0552	0.107	0.0794 ± 0.0212 (27%)
oct-1-en-3-one	2.33	2.38	2.31	2.34 ± 0.03 (1%)
acetic acid	1060000	944000	878000	962000 ± 77100 (8%)
2-methylpropanoic acid	25400	26800	25500	25900 ± 646 (2%)
butanoic acid	6590	6730	5230	6180 ± 676 (11%)
2-methylbutanoic acid	7360	7190	7570	7370 ± 155 (2%)
3-methylbutanoic acid	18100	18000	19100	18400 ± 510 (3%)

[a]SD, standard deviation; CV, coefficient of variation.

Table S9. OAVs of Off-Flavor Compounds in Dried Cocoa Nibs from Costa Rica: Reference Cocoa without Off-Flavor vs. Overfermented Cocoa

odorant	odor activity value (OAV)[a]	
	reference sample	overfermented sample
3-methylphenol	0.25	0.32
4-methylphenol	1.0	0.89
3-ethylphenol	0.51	1.0
4-ethylphenol	0.070	2.0
2-methoxyphenol	56	560
2,6-dimethoxyphenol	0.078	0.10
geosmin	<0.001	<0.001
oct-1-en-3-one	0.79	2.5
acetic acid	6300	2700
2-methylpropanoic acid	34	30
butanoic acid	21	120
2-methylbutanoic acid	75	57
3-methylbutanoic acid	1400	1300

[a]OAVs were calculated as mean concentration/OTC.

Table S10. Concentrations and OAVs of Off-Flavor Compounds in Dried Cocoa Nibs from Ecuador: Reference Cocoa without Off-Flavor vs. Overfermented Cocoa

odorant	OTC[a]	concentration in nibs (µg/ kg)[b]		OAV in nibs[e]	
		reference[c]	overfermented[d]	reference[c]	overfermented[d]
acetic acid	400	1740000	1310000	4400	3300
2-methylpropanoic acid	850	13000	41200	15	48
butanoic acid	52	1210	1550	23	30
2-methylbutanoic acid	130	4010	14600	31	110
3-methylbutanoic acid	14	11600	29000	830	2100

[a]Odor threshold concentration in deodorized cocoa butter; the OTCs were determined using the method detailed in reference 13. [b]Mean values; individual values and standard deviations are available in Tables S11–S12. [c]Reference fermentation following the method commonly practiced on the farm in Ecuador: 500 kg of fermentation mass from a local clone mix, fermented for 5 days in a wooden box with mixing every 24 hours, subsequent sun-drying. [d]Sample taken from the same fermentation box after 10 days of fermentation, subsequent sun-drying. [e]OAVs were calculated as mean concentration/OTC.

Table S11. Concentrations of Off-Flavor Compounds in Dried Nibs of the Reference Cocoa without Off-Flavor from Ecuador

odorant	concentration (µg/kg)			
	experiment 1	experiment 2	experiment 3	mean ± SD (CV)[a]
acetic acid	1780000	1700000	1720000	1740000 ± 31800 (2%)
2-methylpropanoic acid	13400	13200	12400	13000 ± 421 (3%)
butanoic acid	1550	1280	793	1210 ± 313 (26%)
2-methylbutanoic acid	4520	3920	3580	4010 ± 389 (10%)
3-methylbutanoic acid	11400	11900	11300	11600 ± 247 (2%)

[a]SD, standard deviation; CV, coefficient of variation.

Table S12. Concentrations of Off-Flavor Compounds in Dried Nibs of the Overfermented Cocoa from Ecuador

odorant	concentration (µg/kg)			
	experiment 1	experiment 2	experiment 3	mean ± SD (CV)[a]
acetic acid	1470000	1220000	1220000	1310000 ± 118000 (9%)
2-methylpropanoic acid	40200	42900	40600	41200 ± 1180 (3%)
butanoic acid	1580	1250	1810	1550 ± 231 (15%)
2-methylbutanoic acid	14700	14100	15200	14600 ± 419 (3%)
3-methylbutanoic acid	30200	28600	28200	29000 ± 874 (3%)

[a]SD, standard deviation; CV, coefficient of variation.

Table S13. Concentrations and OAVs of Off-Flavor Compounds in Dried Cocoa Nibs from Costa Rica: Second Reference Cocoa without Off-Flavor vs. Cocoa Fermented in a Box with Side Slits

odorant	OTC[a]	concentration in nibs (µg/ kg)[b]		OAV in nibs[e]	
		reference[c]	from box with side slits[d]	reference[c]	from box with side slits[d]
acetic acid	400	2770000	1200000	6900	3000
2-methylpropanoic acid	850	36600	66000	43	78
butanoic acid	52	1150	1100	22	21
2-methylbutanoic acid	130	9250	18800	71	150
3-methylbutanoic acid	14	16400	19400	1200	1400

[a]Odor threshold concentration in deodorized cocoa butter; the OTCs were determined using the method detailed in reference 13. [b]Mean values; individual values and standard deviations are available in Tables S14–S15. [c]Fermentation parameters equalled those applied in the previous reference fermentation in Costa Rica (cf. section 2.3 in the main document and section Fermentation Trials in Costa Rica in the Supporting Information file), i.e., 50 kg of fermentation mass from a local clone mix, fermented for 6 days in a wooden box with mixing every 24 hours, and subsequent sun-drying. [d]Performed in parallel to the second reference fermentation (cf. footnote c) using the same parameters but a box with side slits of approximately 3–5 mm between the cross planks. [e]OAVs were calculated as mean concentration/OTC.

Table S14. Concentrations of Off-Flavor Compounds in Dried Nibs of the Second Reference Cocoa without Off-Flavor from Costa Rica

odorant	concentration (µg/kg)			
	experiment 1	experiment 2	experiment 3	mean ± SD (CV)[a]
acetic acid	2750000	2860000	2720000	2770000 ± 60900 (2%)
2-methylpropanoic acid	33900	40300	35700	36600 ± 2720 (7%)
butanoic acid	1330	1080	1040	1150 ± 129 (11%)
2-methylbutanoic acid	9620	9160	8960	9250 ± 275 (3%)
3-methylbutanoic acid	17700	15900	15600	16400 ± 913 (6%)

[a]SD, standard deviation; CV, coefficient of variation.

Table S15. Concentrations of Off-Flavor Compounds in Dried Nibs of the Cocoa Sample Fermented in a Box with Side Slits from Costa Rica

odorant	concentration (µg/kg)			
	experiment 1	experiment 2	experiment 3	mean ± SD (CV)[a]
acetic acid	1170000	1410000	1010000	1200000 ± 165000 (14%)
2-methylpropanoic acid	64700	66700	66500	66000 ± 902 (1%)
butanoic acid	1190	1130	985	1100 ± 85 (8%)
2-methylbutanoic acid	19200	18900	18400	18800 ± 334 (2%)
3-methylbutanoic acid	19600	19500	19300	19400 ± 130 (1%)

[a]SD, standard deviation; CV, coefficient of variation.
